# At the double for tumor suppressor

**DOI:** 10.7554/eLife.18391

**Published:** 2016-07-15

**Authors:** Mahendra Sonawane

**Affiliations:** Department of Biological Sciences, Tata Institute of Fundamental Research, Mumbai, Indiamahendras@tifr.res.in

**Keywords:** carcinogenesis, Na/K-ATPase, epithelial polarity, Zebrafish

## Abstract

Research on zebrafish reveals how a tumor suppressor works in two different types of cells, and how hypotonic stress promotes tumor formation when the function of this tumor suppressor is lost.

**Related research article** Hatzold J, Beleggia F, Herzig H, Altmüller J, Nürnberg P, Bloch W, Wollnik B, Hammerschmidt M. 2016. Tumor suppression in basal keratinocytes via dual non-cell-autonomous functions of a Na,K-ATPase beta subunit. *eLife* **5**:e14277. doi: 10.7554/eLife.14277

**Image** Epidermal cells in mutant zebrafish display characteristics of cancer during development

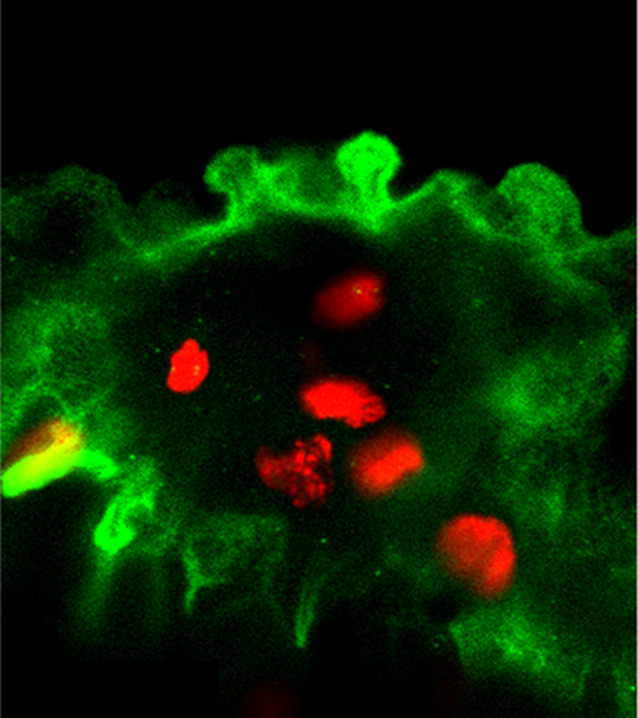

Cell proliferation and growth is strictly controlled in animals. However, when the mechanisms that control these processes break down, the outcomes include tissue hyperplasia and tumor formation. Several genes and pathways involved in the suppression of tumor formation have been identified in the last few decades. Most of these tumor suppressors act within the cells in which they are synthesized, but a small number act on other cells (Chua et al., 2014). Now, in *eLife*, Matthias Hammerschmidt of the University of Cologne and co-workers, including Julia Hatzold as the first author, have shown that a tumor suppressor that is expressed in one layer of the epidermis of developing zebrafish works in this layer and also controls cell polarity and influences growth in the other layer of the epidermis (Hatzold et al., 2016).

Hatzold et al. analyzed a zebrafish mutant called *psoriasis* in which basal epidermal cells exhibit some of the hallmarks of cancers (Hanahan and Weinberg, 2011; Webb et al., 2008). First they showed that a mutation in *atp1b1a* – a gene that encodes the beta subunit of Na,K-ATPase, the enzyme that pumps sodium ions out of cells and potassium ions into cells – caused the epidermal cells to become malignant. Consisting of two subunits, called alpha and beta, Na,K-ATPase also (directly or indirectly) regulates the adhesion of epithelial cells (Vagin et al., 2012). Moreover, researchers have shown that the beta subunit cooperates with a protein called epithelial cadherin to suppress invasiveness (another hallmark of cancer) and to induce the correct polarity in epithelial cells (Rajasekaran et al., 2001)

The zebrafish epidermis has two layers: the inner layer is called the basal epidermis, and the outer layer is called the periderm. In developing zebrafish, *atp1b1a* is expressed in the periderm, the heart and the pronephros (which functions as the kidney in larvae). Consistent with this, Hatzold et al. found that *psoriasis* mutants also exhibited compromised heart and kidney function, leading to hypotonic stress and edema formation (the accumulation of fluid).

Hatzold et al. made three other interesting observations. First, although *atp1b1a* is expressed in the periderm, the malignancy phenotype was actually observed in the basal epidermis. Second, growing the mutants in an isotonic medium to suppress hypotonic stress and edema formation also reduced the number of malignant cells in the basal epidermis. Third, using a poison called ouabain to stop the pumping of ions by Na,K-ATPase led to hypotonic stress and edema formation in wild type zebrafish embryos, but did not result in cell proliferation and malignancy. These observations suggested that the beta subunit of Na,K-ATPase prevents malignancy in the cells of the basal epidermis, and that edema formation (caused by impaired kidney function) makes a substantial contribution to the acquisition of the malignant phenotype (Figure 1).

**Figure 1.**
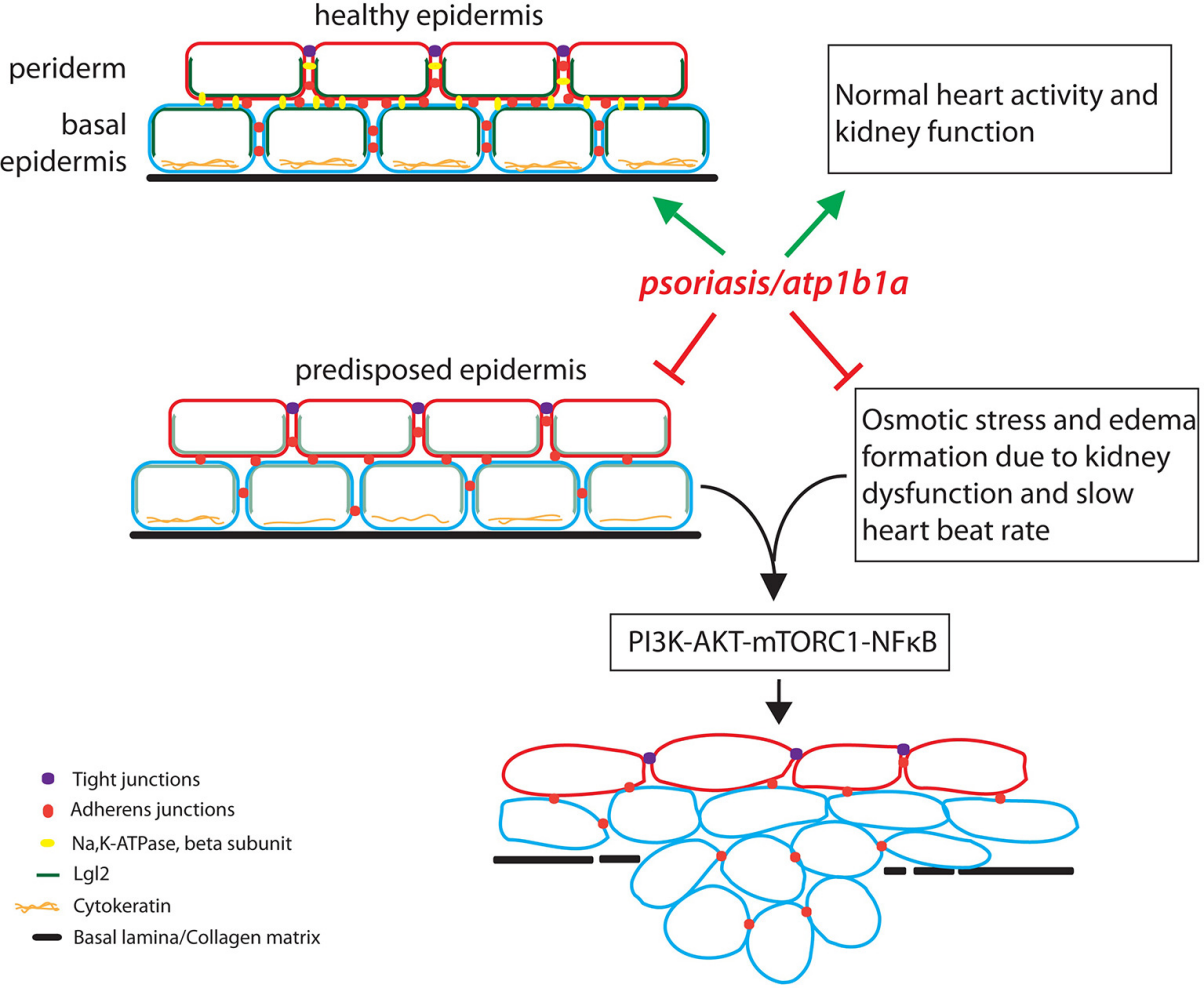
The role of the *atp1b1a* gene in zebrafish. The *atp1b1a* gene is essential (green arrows) for the maintenance of a healthy epidermis (by maintaining the cell polarity and cell adhesion), and for the proper functioning of the heart and pronephros (the larval kidney). The *atp1b1a* function is necessary to suppress the predisposition of the epidermis to malignancy and to prevent hypotonic stress by facilitating normal kidney function (red inhibitory arrows). In the absence of *atp1b1a* function, the predisposed epidermis undergoes a malignant transformation as a consequence of the PI3K-AKT-mTORC1-NFκB pathway being activated by the hypotonic stress caused by a dysfunctional pronephros.

Hatzold et al. performed a further series of experiments to explore the origins of the malignant transformation. In the absence of *atp1b1a* function, the localization of epithelial cadherin (which is involved in cell-cell adhesion and the regulation of cell polarity) and Lgl2 (which ensures that the epithelial cells have the correct polarity; Laprise and Tepass, 2011) – was diminished in both the periderm and the basal epidermis. These effects persisted even in the absence of hypotonic stress and edema formation. Thus, the beta subunit of Na,K-ATPase is responsible for maintaining polarity in both layers of the epidermis, even though it is only expressed in one of these layers. Furthermore, Hatzold et al. showed that the forced expression of *atp1b1a* in the mutant peridermal cells reduced the level of malignancy seen in the basal epidermal cells.

Put together, these lines of evidence point to the fact that the absence of *atp1b1a* function predisposes the cells in the basal epidermis to the malignant phenotypes. However, the acquisition of malignancy depends on hypotonic stress and edema formation, which arise due to impaired functioning of the heart and kidney.

How does the hypotonic stress promote the malignant transformation? Is it simply the mechanical stress generated by the accumulation of fluid below the epidermis, or does the edematous fluid contain factors that promote tumor formation in the predisposed tissue? Going further, does Na,K-ATPase mediate its effect across the epidermal layers via epithelial cadherin (Nelson et al., 2013)? These questions require further investigation.

It remains to be seen how relevant these findings might be in the context of cancer treatment in humans, but they serve to remind us of the wide array of processes and phenomena – from genetic mutations to osmotic stress – that are involved in cancer progression.
